# Incorporation of Blue Honeysuckle Juice into Fermented Goat Milk: Physicochemical, Sensory and Antioxidant Characteristics and In Vitro Gastrointestinal Digestion

**DOI:** 10.3390/foods11193065

**Published:** 2022-10-02

**Authors:** Jiage Ma, Yusi Miao, Jinzhe Li, Yue Ma, Mengguo Wu, Wan Wang, Cong Xu, Zhanmei Jiang, Juncai Hou

**Affiliations:** 1 Key Laboratory of Dairy Science, Ministry of Education, College of Food Science, Northeast Agricultural University, Harbin 150030, China; 2 Heilongjiang Green Food Science Research Institute, Harbin 150028, China

**Keywords:** fermented goat milk, blue honeysuckle juice, antioxidant activity, in vitro digestion

## Abstract

The addition of fruit juice may improve the physicochemical and functional characteristics of dairy products. The study evaluated the effect of 1–6% (*v*/*v*) blue honeysuckle juice (BHJ) on the physicochemical, sensory and antioxidant characteristics of fermented goat milk (FGM) during 21 days of refrigerated storage and in vitro gastrointestinal digestion. The incorporation of BHJ significantly increased (*p* < 0.05) the water-holding capacity, viscosity, redness (a*) value, total phenolic content (TPC) and ferric ion-reducing antioxidant power during storage. Additionally, BHJ affected the microstructure and sensory score of the samples. FGM treated with 4% (*v*/*v*) BHJ exhibited the highest overall acceptability. The supplementation of BHJ diminished the goaty flavor and promoted in vitro protein digestion. Furthermore, the TPC was enhanced in addition to the antioxidant activity of FGM containing BHJ throughout the in vitro digestion. Therefore, FGM supplemented with BHJ serves as a novel and attractive goat dairy product.

## 1. Introduction

Increasing consumer awareness about the health benefits of diet has led to the development of foods with nutritional and functional properties worldwide [1]. Goat milk is widely consumed due to its high nutritional value, digestibility and hypoallergenicity, and represents an excellent delivery vehicle for probiotics and plant-derived polyphenols [2]. Additionally, recent studies report that fermented goat milk (FGM) improves gut microbiota, immunity, metabolic homeostasis and even cognitive growth [3]. Nevertheless, the fragile texture and unpleasant goaty flavor due to the low content of α-S1-casein and high levels of free octanoic acids in FGM limit consumer acceptance of FGM products [4].

To enhance the sensory, nutritional and functional characteristics of dairy, studies have explored the feasibility of incorporating natural products, such as jujube pulp, strawberry juice, elderberry juice and rose flower extract [5,6,7]. Further, yogurt supplemented with polyphenol-rich plant extracts exhibits improved biological activities, including antioxidant and angiotensin-converting enzyme inhibitory and antibacterial activity, in addition to the strong viability of probiotics in the dairy products [8]. Therefore, it is of interest to improve the quality attributes and functionalities of FGM and develop new products with potential health benefits by adding fruits or their derivatives.

Blue honeysuckle (*Lonicera caerulea* L.), belonging to family Caprifoliaceae, is widely harvested in Northeast China, Russia, Korea and Japan [9]. Blue honeysuckle fruits have attracted increased attention not only for their pleasant flavor, but also because of their abundance of nutritional ingredients, especially phenolic acids, flavonoids and anthocyanins [10]. Numerous investigations have demonstrated the strong affinity of phenolic compounds in fermented dairy products with amino, carboxyl and hydroxyl groups of proteins and peptides via covalent and noncovalent bonds, which alter their structure and properties, resulting in significantly enhanced bioavailability of polyphenols [11]. Notably, proteins with high molecular weight may be digested into small bioactive peptides and amino acids with antioxidant activity during simulated gastrointestinal digestion, which may lead to the release of phenolics bound to these proteins, thereby enhancing their antioxidant attributes [12]. Furthermore, dairy products carry plant-derived bioactive substances such as polyphenols, protecting them from adverse environmental conditions in the digestive tract and transporting them to the small intestine and colon. However, as far as we know, the impact of phenolic-rich blue honeysuckle juice (BHJ) on the quality and beneficial bioactivities of FGM products has received limited attention [13,14].

Therefore, the work presented here reports the effects of supplementation with BHJ at different concentrations (1%, 2%, 4% and 6%) on the physicochemical characteristics of FGM during 21 days of storage at 4 °C based on pH, water-holding capacity (WHC) and viscosity. The appearance, microstructure, sensory acceptance and antioxidant activity were also evaluated during the refrigerated storage. Additionally, the nutritional and functional properties of FGM treated with BHJ in terms of protein digestibility, total phenolic content (TPC) as well as antioxidant activity were systematically assessed during each step of in vitro simulated digestion.

## 2. Materials and Methods

### 2.1. Materials

Goat milk was purchased from Heilongjiang Province, China. Blue honeysuckle fruits were obtained from a local supermarket (Qitaihe, China). Freeze-dried kefir mild 01 commercial starter culture (*Streptococcus thermophilus*, *Leuconostoc mesenteroides* ssp. *mesenteroides*, *Lactobacillus acidophilus, Lactococcus lactis* ssp. *lactis*, *Lc. Lactis* ssp. *Cremoris* and *Lc. lactis* ssp. *lactis* biovar *diacetylactis*) was acquired from Danisco (Beijing, China). 2,2-Diphenyl-1-picryl-hydrazyl (DPPH), 2,4,6-tripyridyl-s-triazine (TPTZ) and (±)-6-hydroxyl-2,5,7,8-tetramethyl-2-carboxylic acid (Trolox) were bought from Biotopped Technology Co., Ltd. (Beijing, China). Nile Red and Nile Blue were purchased from Yuanye Biotechnology Co., Ltd. (Shanghai, China). Sterile distilled water was utilized throughout the experiment. All other chemicals used were of analytical grade.

### 2.2. Preparation of FGM Supplemented with Blue Honeysuckle Juice

The blue honeysuckle fruit was first washed with sterile distilled water and ground into BHJ using a tabletop juicer. Next, the BHJ was filtered using a 300-mesh sieve to remove the dross and peel. The remaining BHJ was evaporated at 45 °C to 15 degrees Brix (°Bx) with a rotary evaporator. The concentrated BHJ was pasteurized at 65 °C for 30 min. Simultaneously, fresh goat milk was filtered to remove impurities and supplemented with 0.6% β-cyclodextrin, 0.9% lactitol, 1% goat milk extract and 7% sugar (*v*/*v*) to reduce goaty flavor. BHJ was blended into the goat milk to obtain 1%, 2%, 4% and 6% (*v*/*v*) concentrations. Mixtures were prepared using a magnetic stirrer (Wiggens, Germany) and then homogenized under 20 MPa, followed by pasteurization at 65 °C for 30 min. Thereafter, all mixtures were cooled to 28 °C and inoculated with 0.01% (*w*/*v*) kefir mild 01 starter culture. Mixed samples were fermented at 28 °C for 10 h, then cooled to 4 °C and stored for 24 h. Plain FGM served as the control. All the FGM products were prepared in triplicate. Finally, FGM samples with or without BHJ were monitored at 4 °C during 0, 7, 14 and 21 days of storage.

### 2.3. Determination of pH, WHC and Viscosity

The pH value was recorded with a PB-10 pH meter (Sartorius, Beijing, China). The WHC was analyzed via centrifugation at 4000× *g* for 10 min according to Li et al. [15]. The supernatant was immediately discarded and WHC (%) was calculated as the ratio of the residual pellet weight to the original weight before centrifugation. Viscosity was measured with a HAAKE MARS 40 rotational rheometer (Thermo Fisher Scientific Inc., Harbin, China) using a parallel plate (diameter 35 mm, gap 0.05 mm). The apparent viscosity of plain and mixed samples was determined at 25 °C in the shear rate ($\dot{\gamma }$) range from 0.1 to 100 s^−1^ [16].

### 2.4. Color Measurement

The color was assessed with a ZE6000 colorimeter (NIPPON DENSHOKU, Osaka, Japan). A white tile calibration plate was used to calibrate the colorimeter before color measurements. The lightness (L*), redness (a*) and yellowness (b*) of the FGM with or without BHJ in an optically flat glass dish were evaluated in triplicate. The total color difference (ΔE*) was calculated as follows:

(1) $$\Delta E*=\sqrt{\Delta L^{*}{}^{2}+\Delta a^{*}{}^{2}+\Delta b^{*}{}^{2}}$$

where identifier “∆” represents the difference of the values after and before different treatments.

### 2.5. Total Polyphenol Contents and Antioxidant Properties

The aqueous extraction procedure was carried out in a mixture of 5 mL FGM with or without BHJ and 25 mL methanol solution (75%), as described by Öztürk et al. [17]. The resulting mixtures were swirled for 1 min and then treated with ultrasound at 4 °C for 15 min. The mixture was centrifuged at 5000× *g* at 4 °C for 10 min. The supernatant was obtained and stored at 4 °C for further analysis. The total polyphenol content (TPC) was analyzed using the Folin–Ciocalteu method [18]. Gallic acid was used as the standard. The above extract (1.0 mL) was mixed with methanol (1.0 mL) and distilled water (5 mL). After incubation with 0.5 mL diluted 1:1-fold Folin–Ciocalteu reagent for 5 min, 1.0 mL Na_2_CO_3_ (5%, *w*/*v*) was added and thoroughly mixed. Subsequently, the mixtures were incubated at 25 °C for 60 min and the optical density at 725 nm was detected. Results were expressed as microgram gallic acid equivalents (GAE) per gram of samples.

The determination of ferric ion-reducing antioxidant power (FRAP) was carried out as previously published [19], with some modifications. The sample extract (0.1 mL) was mixed with methanol (0.9 mL) and 3 mL of FRAP working solution and incubated at 37 °C for 30 min. The absorbance of the mixtures was evaluated at 593 nm. According to the standard curve of Trolox, the FRAP content of the sample was expressed as Trolox content per liter of sample (μmol Trolox·L^−1^). Meanwhile, the 2,2-Diphenyl-1-picryl-hydrazyl (DPPH) free-radical scavenging activity was assessed [20]. The extract (0.5 mL) was evenly mixed with methanol (0.5 mL) and 0.2 mmol/L DPPH solution (2.0 mL). The resultant mixture was reacted for 30 min at 25 °C in the dark and the absorbance was detected at 517 nm. The results were expressed as micromoles of Trolox per gram of sample (μmol Trolox·g^−1^).

### 2.6. Microstructure Analysis

The microstructure was analyzed via ultrahigh distraction microscopy (Deltavision OMX SR, GE, Boston, MA, USA) [21]. FGM sample with or without BHJ (1 mL) diluted 15-fold was stained with 0.1% Nile Red (20 μL) and 1% Nile Blue (25 μL), respectively, in dark for 30 min. The sample (1.5 μL) was analyzed at the excitation wavelength of 530 nm (protein distribution) and 497 nm (fat distribution), respectively.

### 2.7. Sensory Evaluation

The sensory evaluation was performed after 21 days of storage according to Ardabilchi Marand et al. [22], with some modification. Twelve trained panelists (50% male and 50% female, 18–35 years old) were randomly recruited. The panel was trained for 8 h over one month (2 h/week). The FGM samples were analyzed three times in a random presentation order among these panelists in sensory evaluation laboratory. Sensory analysis, including appearance (color and whey-off), texture (consistency, gel strength and mouthfeel), flavor (aroma and odor), taste (sweet, sour and bitter) and overall acceptability of FGM samples was based on 5-point hedonic scale ranging from 1 (extremely dislike) to 5 (extremely like).

### 2.8. In Vitro Gastrointestinal Digestion

An in vitro digestion model was established as previously described [23,24], with minor modification. Regarding the oral step, FGM samples with or without BHJ were mixed homogeneously with artificial saliva (1:1, *v*/*v*) at 37 °C for 30 s. Subsequently, the digested samples were mixed uniformly with simulated gastric fluids (1:1, *v*/*v*), and the pH value was adjusted to 2.0 before treatment with 3000 U/mL porcine pepsin. The samples with or without BHJ were then shaken at 100 rpm for 60 min at 37 °C. Thereafter, the mixture with or without BHJ was adjusted to pH 7.0 and was treated with intestinal phase (1:1, *v*/*v*) containing 250 U/mL pancreatin and 12 mg/mL bile salts. Aliquots of the digesta were collected from the oral, gastric and intestinal phase at different intervals (0, 20, 40, 60, 80 and 100 min) and the supernatants were stored at −80 °C for further analysis. Meanwhile, the in vitro protein digestibility was determined using the BCA method and 15% trichloroacetic acid (TCA) was added to terminate the simulated gastric and intestinal digestion [25]. The supernatants were collected via centrifugation at 8000× *g* for 15 min and the protein digestibility (%) was calculated as the ratio of nitrogen in supernatant to nitrogen in undigested sample × 100.

### 2.9. Statistical Analysis

All of the determinations were performed for three times and the data were reported as mean ± standard deviation (SD). One-way analysis of variance (ANOVA) was performed using SPSS 26.0 software (SPSS Inc., Chicago, IL, USA). Fisher’s least significant difference (LSD) test was used to compare means. Differences were considered statistically significant at *p* < 0.05.

## 3. Results and Discussions

### 3.1. Physicochemical Properties of FGM Supplemented with BHJ during Storage

**Figure 1:**
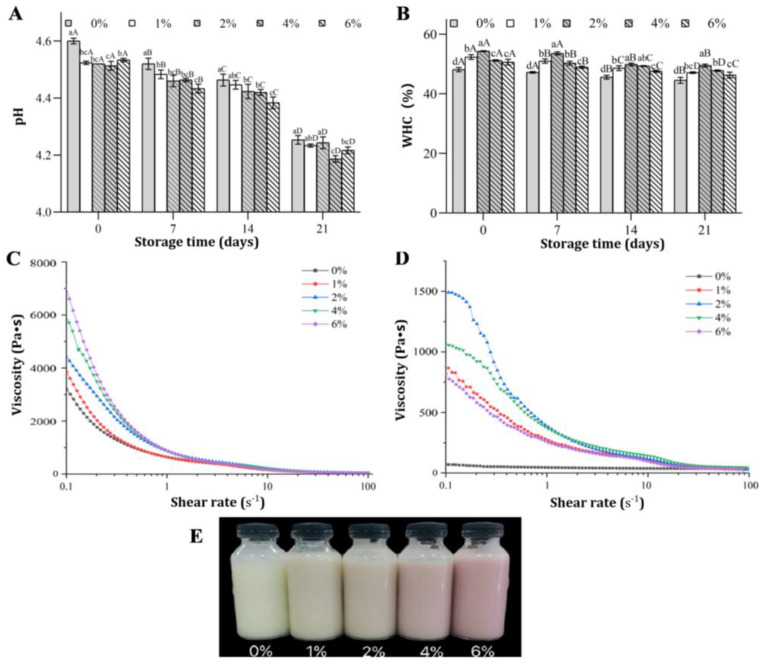
Physicochemical properties of fermented goat milk as affected by various blue honeysuckle juice concentration. (**A**) pH value; (**B**) water-holding capacity; (**C**) viscosity on day 0 and (**D**) after 21 days of storage at 4 °C; (**E**) visual appearance. Note: Different lowercase letters denote significant differences (*p* < 0.05) between different concentrations of blue honeysuckle juice on the same day; different uppercase letters denote significant differences (*p* < 0.05) between different storage times at the same levels of blue honeysuckle juice concentrations.

The pH value, WHC and viscosity of FGM supplemented with BHJ during 21 days of storage at 4 °C were evaluated (Figure 1). The pH of all samples was remarkably reduced during the storage (Figure 1A). Similar results were reported in a previous study of jujube pulp-enriched yogurts [26]. The pH value of unsupplemented FGM was significantly higher than that of FGM fermented with BHJ on day 7 (*p* < 0.05). No significant variation in pH was found compared with the control when BHJ concentrations of 1% and 2% were used after storage for 21 days at 4 °C (*p* > 0.05). Meanwhile, the pH of FGM supplemented with 4% and 6% BHJ was significantly lower than that of the control group after 21 days of storage (*p* < 0.05). As shown in Figure 1B, the WHC of FGM treated with 2% BHJ was 54.30 ± 0.17%, which was significantly higher than that of the other groups, while the WHC of the control was the lowest at 48.14 ± 0.37% before storage (*p* < 0.05). Further, the highest WHC was detected in FGM containing 2% BHJ at 0, 7 and 21 days of storage. Overall, the WHC was increased with increase in BHJ concentration up to 2%. However, any further increase in BHJ concentration weakened its WHC, probably due to the interaction between polyphenols in BHJ and casein in FGM, which further altered the protein conformation and affinity, and enhanced the gel strength of FGM, thereby increasing its WHC [27]. Nevertheless, excessive levels of BHJ disrupted the excessive crosslinking and gel network, which decreased its capacity to hold whey, thereby reducing the WHC.

The rheological behavior of FGM formulated with various concentrations of BHJ during the initial stages of the storage period (0 d) is presented in Figure 1C. Generally, all samples exhibited shear thinning behavior of a typical non-Newtonian fluid. Increasing the concentration of BHJ significantly improved the apparent viscosity (*p* < 0.05), and the FGM sample supplemented with 6% BHJ displayed the highest viscosity compared with the control (without BHJ). The greater viscosity may be related to the interaction between FGM and fiber, protein and soluble solids in concentrated BHJ, which formed a stronger three-dimensional network and increased the flow resistance of FGM [28,29]. This also revealed that the viscosity depends on composition, starter formulation and processing method [30]. Additionally, an obvious reduction in viscosity was observed in all FGM after 21 days of storage (Figure 1D). The FGM containing 2% BHJ exhibited the highest viscosity, followed by FGM supplemented with 4% BHJ. A similar finding was reported by Cais-Sokolinska and Walkowiak-Tomczak [6], suggesting that the viscosity of yogurt decreased following the addition of 21.25% restructured elderberry juice after storage, probably due to the degradation of the FGM network and the liquefaction of gels by kefir starter cultures [31]. Consequently, the incorporation of moderate BHJ in FGM enhances the physicochemical properties by increasing the WHC and viscosity of FGM.

### 3.2. Color of FGM Supplemented with BHJ during Storage

Color is one of the most vital visual attributes determining consumer acceptance and willingness to pay for food [32]. The color of FGM samples following the incorporation of BHJ is shown in Table 1 and Figure 1E. It was conspicuous that the L* and b* of FGM decreased significantly with increasing concentration of BHJ, while a significant opposite trend for the a* value was observed (*p* < 0.05). A previous study showed that the changes in the color of anthocyanin could be related to different chemical structures as a function of the pH value, such as the formation of flavonoid cations (red) mainly under strong acid conditions [33]. Additionally, the ΔE* values of FGM supplemented with different concentrations of BHJ were significantly higher (*p* < 0.05) than control samples without added BHJ. The results revealed that the incorporation of BHJ caused color changes in FGM. Furthermore, no significant changes were detected in the color parameters of FGM supplemented with similar concentrations of BHJ under prolonged storage (*p* > 0.05).

### 3.3. Total Phenolic Content and Antioxidant Activity of FGM Supplemented with BHJ during Storage

**Figure 2:**
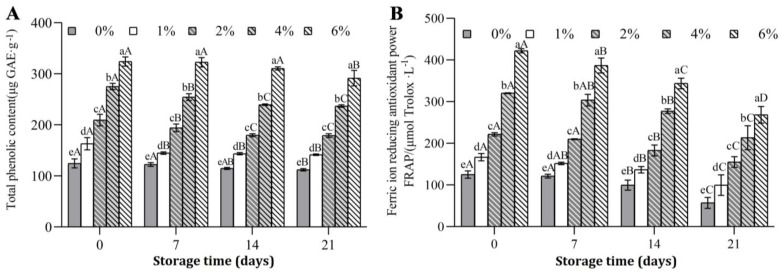
Total phenolic content (**A**) and antioxidant activity (**B**) of FGM supplemented with BHJ during 21 days of storage at 4 °C. Note: Different lowercase letters denote significant differences (*p* < 0.05) between different concentrations of blue honeysuckle juice; different uppercase letters denote significant differences (*p* < 0.05) between different storage times.

The TPC and FRAP levels of different experimental groups are depicted in Figure 2. As shown in Figure 2A, the addition of BHJ rich in polyphenols to FGM significantly enhanced the TPC compared with the controls (*p* < 0.05). Before storage, the TPC of 6% BHJ-enriched FGM (324.24 ± 9.01 μg GAE·g^−1^) was approximately 2.6-fold higher than that of the control (124.59 ± 8.89 μg GAE·g^−1^). Further, the FRAP of FGM was also notably affected by BHJ supplementation in a dose-dependent manner, with a similar trend observed for TPC (Figure 2B). At the end of 21 days of storage at 4 °C, the FRAP of the control group (57.24 ± 13.08 μmol Trolox·L^−1^) was significantly lower than that of all groups treated with BHJ, while the FRAP of FGM supplemented with 6% BHJ (268.62 ± 19.95 μmol Trolox·L^−1^) was significantly higher than in other experimental groups, which was 4.69-fold that of the control group (*p* < 0.05). Notably, the TPC and FRAP of all FGM types declined with extended storage, which may be related to the formation of complexes between BHJ polyphenols and FGM proteins [33]. Additionally, the changes in pH values during storage may promote the decomposition of anthocyanins, thereby reducing the antioxidant activity of FGM. The result is also consistent with previous findings that the incorporation of anthocyanin-rich riceberry rice increased the FRAP values of yogurt, whereas the reduced a* value, anthocyanin content and antioxidant capacity of yogurt were detected during 21 days of storage [34].

### 3.4. Sensory Analysis of FGM Supplemented with BHJ

The sensory scores for different FGM samples based on the 5-point hedonic scale after 21 days of refrigeration are summarized in Table 2. The concentration of BHJ (2–4%, *v*/*v*) had a statistically significant effect on the appearance, texture and flavor of FGM (*p* < 0.05), without a significant impact on taste (*p* > 0.05). Additionally, the sensory characteristics reflected physicochemical parameters. In terms of color, goat milk blended with an appropriate amount of BHJ appears pinkish-purple, which stimulates consumer appetite and purchase intentions. Nevertheless, the lowest scores were detected when FGM was supplemented with 6% BHJ (*p* < 0.05), which was predictable due to reduced scores for the whey-off of FGM samples treated with excessive BHJ, as well as the unacceptable odor and taste. FGM supplemented with 4% BHJ was associated with the highest overall acceptability, indicating that the formulation effectively masked and improved the goaty flavor of FGM (*p* < 0.05).

### 3.5. Protein Digestibility of FGM Supplemented with BHJ during Simulated In Vitro Digestion

**Figure 3:**
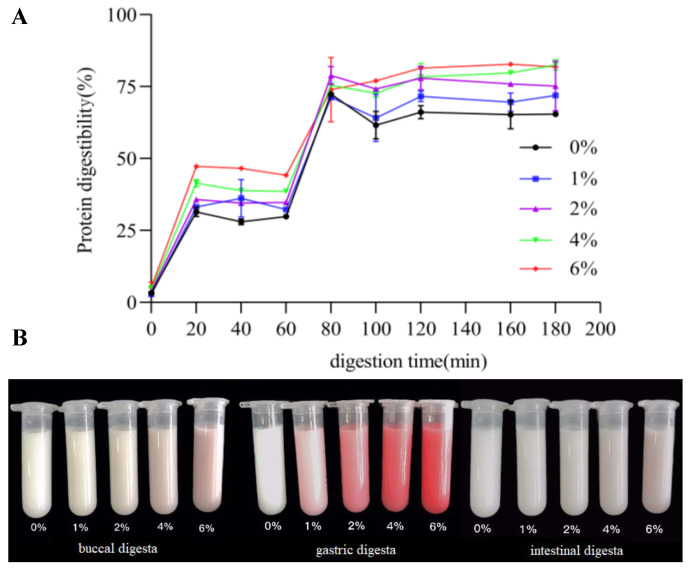
Protein digestibility (**A**) and visual appearance (**B**) of FGM supplemented with BHJ during in vitro digestion.

The protein digestibility of FGM is affected by various BHJ concentrations during simulated in vitro digestion, as shown in Figure 3. At the end of oral digestion (0 min), the protein digestibility of the control group and the FGM supplemented with 6% BHJ was (3.18 ± 0.41)% and (5.26 ± 1.19)%, respectively (Figure 3A). At the beginning of the gastric phase (0–20 min), the protein digestibility of FGM samples increased sharply and ranged between 25% and 50%. At the end of the gastric phase (60 min), the incorporation of 1–6% BHJ enriched with phenolics markedly increased the in vitro protein digestibility of FGM when compared with the control (29.93 ± 0.53)%, probably due to the hydrolysis of casein and whey protein by pepsin, resulting in large peptides [35]. After 20 min of intestinal digestion, the protein digestibility was again dramatically increased by about 30%. At the end of the intestinal digestion (60 to 80 min), there was no remarkable variation between the protein digestibility of FGM supplemented with 4% and 6% BHJ (*p* > 0.05), while both were significantly higher than that of the control (65.31 ± 0.28)% (*p* < 0.05), indicating rapid hydrolysis of FGM protein and the production of smaller low-molecular-weight peptides during intestinal digestion.

The visual appearance of digesta of FGM treated with different concentrations of BHJ after buccal, gastric and intestinal digestion in vitro is depicted in Figure 3B. This phenomenon may be due to fact that the goat milk proteins consist of casein with a loose and highly flexible structure, and whey protein shows a spherical three-dimensional structure. Structural differences of goat milk proteins in FGM added with BHJ may significantly affect their degradation and transformation in the gastrointestinal tract, especially their sensitivity to digestive enzymes [34]. The results were consistent with those of Cao and Xiong [36], who reported that the presence of epigallocatechin gallate under acidic and neutral pH conditions induced structural unfolding and greater digestibility of whey protein isolate. A previous study also demonstrated that the incorporation of chlorogenic acid improved the digestibility and functionality of both casein and whey protein mediated via noncovalent interaction of milk proteins [37]. The structural modification of the protein due to the formation of polyphenol–protein complexes increased the access to enzymatic sites, resulting in functional enhancement [38].

### 3.6. Microstructure of FGM Supplemented with BHJ during Storage and In Vitro Digestion

**Figure 4:**
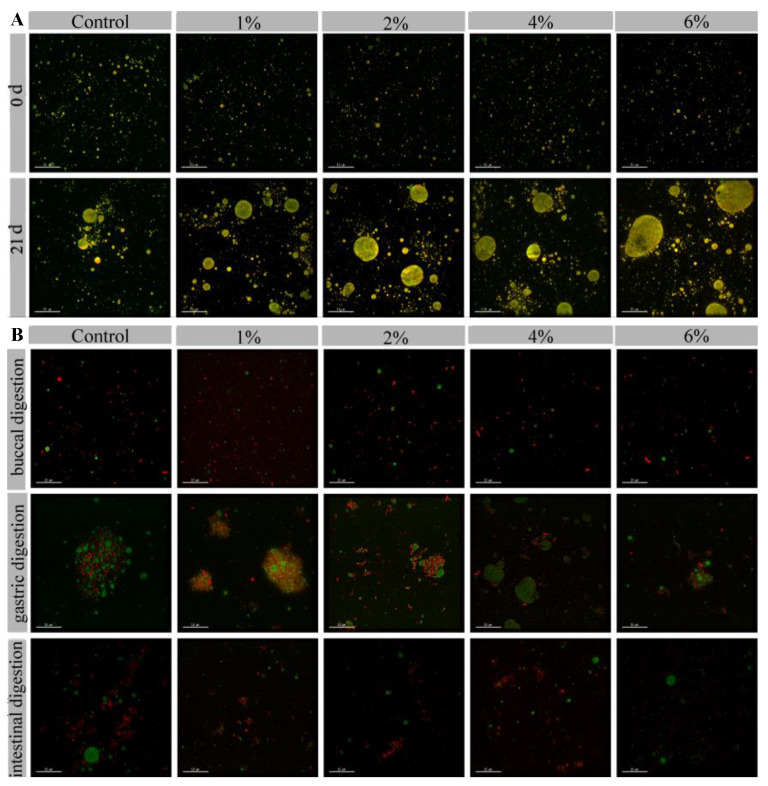
Microstructure of FGM supplemented with BHJ during storage on day 0 and after 21 days at 4 °C (**A**) and during in vitro buccal, gastric and intestinal digestion (**B**) (green denotes fat and red indicates protein).

The microstructure of FGM supplemented with BHJ during storage at 4 °C and during digestion are illustrated in Figure 4 using an ultrahigh-resolution optical microscope. At the beginning of storage (0 d), all FGM formulations presented an evenly distributed and tightly compact structure, suggesting the relatively homogeneous droplets and protein network of FGM samples (Figure 4A). The denser and more uniform the microstructure, the stronger the gel strength and the better the quality of the yogurt [39]. After storage at 4 °C for 21 days, all FGM samples formed large aggregates. Further, the diameter of the aggregates gradually increased with the increase in the concentration of BHJ. Specifically, the FGM containing 6% BHJ exhibited a larger aggregation structure, with many droplets measuring approximately 15–20 μm in diameter, due to the decrease in the negative charge of the κ-casein hairy layer around the casein micelles and the collapse onto the surface of the micelles, resulting in reduced spatial repulsion between the casein micelles and aggregation when BHJ containing a large amount of organic acids such as citric acid and malic acid was added to FGM as an acidifier [40].

The microstructure of the FGM samples during in vitro buccal, gastric and intestinal digestion was evaluated (Figure 4B). At the end of buccal digestion, the protein network was evenly distributed. The large protein aggregates in the gastric digesta and the fat globules were uniformly distributed within the FGM protein matrix. Higher levels of BHJ reduced the degree of flocculation. The results were similar to protein crosslinking to form hollow three-dimensional skeletons containing bright green dots representing fat [41]. Furthermore, the intestinal digestion facilitated the formation of fat globules of different sizes and fewer protein aggregates, which may interfere with enzymatic digestion, resulting in reduced bioavailability of FGM samples. Additionally, the flocculation of FGM was weakened and the dispersion was uniform with the increased concentration of BHJ. The differences in the microstructure of FGM samples supplemented with various concentrations of BHJ can be attributed to the interactions between milk protein and polyphenols derived from BHJ [42].

### 3.7. TPC and Antioxidant Activity of FGM Supplemented with BHJ during In Vitro Digestion

**Figure 5:**
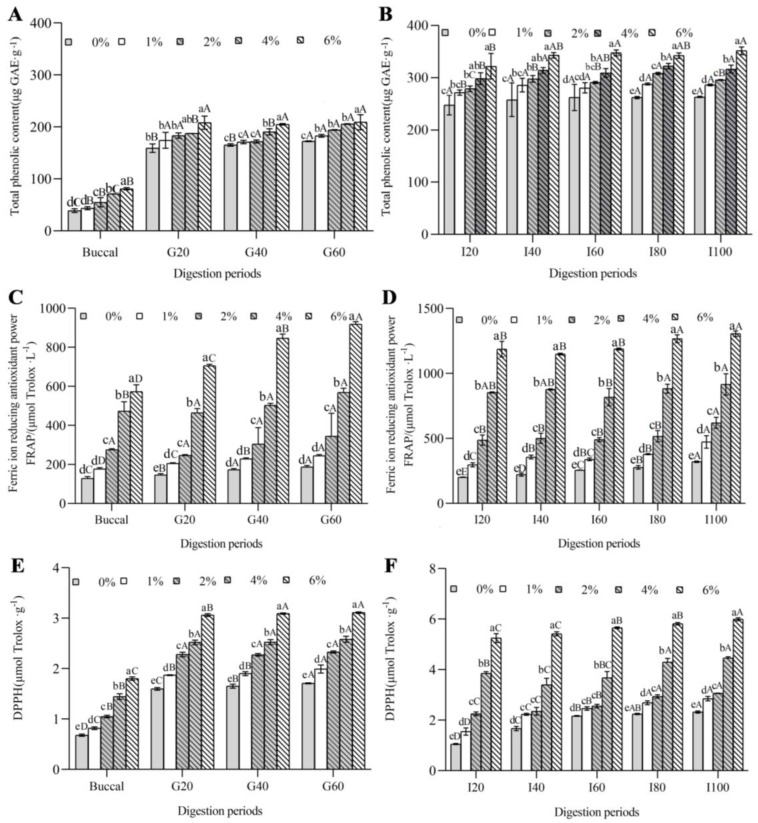
Total phenolic content (**A**,**B**) and antioxidant activity measured by FRAP (**C**,**D**) and DPPH (**E**,**F**) of FGM supplemented with BHJ after in vitro buccal, gastric and intestinal digestion: Buccal digestion refers to the buccal phase for 0.5 minutes; G20, G40 and G60 indicate the gastric phases for 20, 40 and 60 min, respectively; I20, I40, I60, I80 and I100 indicate the intestinal phases lasting 20, 40, 60, 80 and 100 min, respectively. Note: Different lowercase letters denote significant differences (*p* < 0.05) between different concentrations of blue honeysuckle juice; different uppercase letters denote significant differences (*p* < 0.05) between different digestion periods.

The TPC and antioxidant activity of FGM incorporated with different concentrations of BHJ were monitored under simulated in vitro digestion (Figure 5). Overall, the TPC, FRAP and DPPH free-radical scavenging activity of all FGM formulations were gradually increased during the in vitro digestion. At the end of buccal digestion, the TPC of the plain FGM (control) was (38.73 ± 3.61) μg GAE·g^−1^, which was not significantly different from that of FGM supplemented with 1% BHJ (Figure 5A). Meanwhile, the TPCs of FGM treated with 2%, 4% and 6% BHJ were significantly higher than that of the control (*p* < 0.05). At the end of gastric digestion, the highest TPC was observed in FGM supplemented with 6% BHJ, which was 1.65-fold higher than the TPC of the control group. Additionally, the TPC of plain FGM was significantly lower than that of FGM supplemented with different BHJ concentrations at the end of the intestinal digestion (*p* < 0.05), and the TPC of FGM with 6% BHJ was up to (351.55 ± 7.42) μg GAE·g^−1^, which was 34% higher than that of the control (Figure 5B), which can be explained by the digestion facilitating the release of phenolic compounds from the dairy matrix [43]. Other studies reported that phenolics inhibited lipase activity and fat absorption. Further food delivery and casein hydrolysis in the gastrointestinal tract contributed to the release of phenolics, which altered the pH from an acidic to mild-alkaline level, resulting in the separation of proton from the hydroxyl group on the aromatic ring of phenols [44,45]. Moreover, the hydrolysis and conformational changes of milk protein may play a crucial role in the release of polyphenols.

Regarding FRAP, it is interesting to note that all samples showed an upward trend during digestion (Figure 5C,D). The highest FRAP value of (1305.04 ± 21.65) μmol Trolox·L^−1^ was displayed by FGM supplemented with 6% BHJ during all the phases of digestion when compared with the control (*p* < 0.05). In DPPH terms, all FGM formulations were remarkably affected by BHJ concentration and the addition of BHJ to FGM increased DPPH values significantly than control, which was consistent with the results of FRAP (*p* < 0.05) (Figure 5E,F). At the end of the intestinal phase, the DPPH free-radical scavenging activity of FGM incorporated with 6% BHJ reached the highest value of (5.99 ± 0.06) μmol Trolox·g^−1^, which was 2.58-fold higher than that of the control. Similar increases in antioxidant activities were reported previously [38]. It has been reported that there was a positive correlation between TPC and FRAP or DPPH during simulated gastrointestinal digestion of food products containing polyphenols [46]. Enhanced FRAP or DPPH values could be explained by the release of antioxidant peptides and free amino acids from the milk protein [42].

## 4. Conclusions

The incorporation of BHJ had a positive effect on some of the sensory, nutritional and functional characteristics of FGM made with kefir during the 21 days of storage and evaluation. Compared with FGM produced without BHJ (control), FGM supplemented with 1–6% (*v*/*v*) BHJ showed higher WHC, a* value, apparent viscosity, TPC and antioxidant activity based on FRAP with larger aggregated fat globules. Further, the WHC, pH value and viscosity of all formulations declined with extended storage. The addition of 4% BHJ to FGM resulted in an attractive color, weak goaty flavor and the highest sensory quality. At the end of in vitro simulated gastrointestinal digestion, the BHJ addition resulted in a significantly improved digestion of proteins, TPC, FRAP and DPPH radical scavenging ability. Additionally, a more uniform and less flocculated microstructure of digesta in BHJ-added samples was observed during the gastric and intestinal digestion. The overall findings indicate that BHJ is a potential and promising natural ingredient for development of novel dairy products with multifunctional characteristics.

## Figures and Tables

**Table 1:** Color parameters of FGM supplemented with BHJ during refrigerated storage.

Color Parameters	Storage Time (Days)	0% (Control)	1% BHJ	2% BHJ	4% BHJ	6% BHJ
L*	0	86.09 ± 0.17 ^aA^	82.49 ± 0.12 ^bA^	82.77 ± 0.21 ^cA^	81.68 ± 0.38 ^dA^	77.90 ± 0.33 ^eA^
	7	86.33 ± 0.18 ^aA^	82.47 ± 0.19 ^bA^	82.41 ± 0.24 ^cA^	81.10 ± 0.76 ^dA^	77.45 ± 0.08 ^eA^
	14	86.47 ± 0.38 ^aA^	82.85 ± 0.79 ^bA^	82.81 ± 0.12 ^cA^	81.50 ± 0.11 ^dA^	77.90 ± 0.31 ^eA^
	21	86.32 ± 0.11 ^aA^	82.54 ± 0.08 ^bA^	82.26 ± 0.17 ^cA^	81.82 ± 0.12 ^dA^	77.84 ± 0.03 ^eA^
a*	0	−1.72 ± 0.19 ^eA^	2.43 ± 0.03 ^dA^	4.85 ± 0.05 ^cA^	8.30 ± 0.46 ^bA^	10.89 ± 0.04 ^aA^
	7	−1.73 ± 0.02 ^eA^	2.44 ± 0.07 ^dA^	4.87 ± 0.07 ^cA^	8.29 ± 0.18 ^bA^	10.88 ± 0.02 ^aA^
	14	−1.72 ± 0.03 ^eA^	2.43 ± 0.05 ^dA^	4.86 ± 0.15 ^cA^	8.29 ± 0.13 ^bA^	10.89 ± 0.09 ^aA^
	21	−1.72 ± 0.09 ^eA^	2.44 ± 0.33 ^dA^	4.85 ± 0.06 ^cA^	8.28 ± 0.05 ^bA^	10.88 ± 0.14 ^aA^
b*	0	5.55 ± 0.22 ^aA^	4.82 ± 0.14 ^bA^	3.71 ± 0.03 ^cA^	2.65 ± 0.11 ^dA^	1.78 ± 0.02 ^eA^
	7	5.53 ± 0.01 ^aA^	4.81 ± 0.05 ^bA^	3.73 ± 0.06 ^cA^	2.68 ± 0.21 ^dA^	1.76 ± 0.03 ^eA^
	14	5.54 ± 0.12 ^aA^	4.83 ± 0.06 ^bA^	3.70 ± 0.21 ^cA^	2.62 ± 0.33 ^dA^	1.79 ± 0.11 ^eA^
	21	5.55 ± 0.23 ^aA^	4.82 ± 0.05 ^bA^	3.70 ± 0.02 ^cA^	2.68 ± 0.02 ^dA^	1.77 ± 0.02 ^eA^
ΔE*	0	—	5.54	7.59	11.33	15.50
	7	—	5.73	7.88	11.66	15.88
	14	—	5.55	7.75	11.55	15.70
	21	—	5.67	7.94	11.34	15.65

Note: Different lowercase letters in the same row denote significant differences (*p* < 0.05); different uppercase letters in the same column denote significant differences (*p* < 0.05).

**Table 2:** Sensory attributes of fermented goat milk (FGM) as affected by various blue honeysuckle juice (BHJ) concentration (*v*/*v*) based on 5-point hedonic scale.

Attribute	0% BHJ (Control)	1% BHJ	2% BHJ	4% BHJ	6% BHJ
Apparence	4.04 ± 0.15 ^b^	4.35 ± 0.31 ^ab^	4.56 ± 0.17 ^a^	4.54 ± 0.22 ^a^	3.67 ± 0.24 ^c^
Texture	3.73 ± 0.32 ^b^	4.23 ± 0.55 ^ab^	4.63 ± 0.33 ^a^	4.51 ± 0.43 ^a^	3.02 ± 0.14 ^c^
Flavor	3.68 ± 0.28 ^c^	4.15 ± 0.47 ^bc^	4.50 ± 0.21 ^ab^	4.83 ± 0.16 ^a^	3.05 ± 0.27 ^d^
Taste	4.28 ± 0.30 ^a^	4.28 ± 0.19 ^a^	4.37 ± 0.43 ^a^	4.50 ± 0.41 ^a^	3.39 ± 0.48 ^b^
Overall Acceptability	3.93 ± 0.21 ^c^	4.25 ± 0.07 ^b^	4.51 ± 0.13 ^ab^	4.60 ± 0.17 ^a^	3.28 ± 0.23 ^d^

Note: Different lowercase letters in the same row denote significant differences (*p* < 0.05).

## Data Availability

Data are contained within the article.
